# Efficacy of a dilemma-focused intervention for unipolar depression: study protocol for a multicenter randomized controlled trial

**DOI:** 10.1186/1745-6215-14-144

**Published:** 2013-05-17

**Authors:** Guillem Feixas, Arturo Bados, Eugeni García-Grau, Adrián Montesano, Gloria Dada, Victoria Compañ, Mari Aguilera, Marta Salla, Joan Miquel Soldevilla, Adriana Trujillo, Clara Paz, Lluís Botella, Sergi Corbella, Luis Ángel Saúl-Gutiérrez, José Cañete, Miquel Gasol, Montserrat Ibarra, Leticia Medeiros-Ferreira, José Soriano, Eugénia Ribeiro, Franz Caspar, David Winter

**Affiliations:** 1 Department of Personality, Assessment and Psychological Treatments, Faculty of Psychology, Universitat de Barcelona, Campus Mundet, Passeig Vall Hebron, 171, Barcelona, Spain; 2 Institut de Recerca Cervell, Cognició i Conducta (IR3C), Universitat de Barcelona, Campus Mundet, Passeig Vall Hebron, 171, Barcelona, Spain; 3 Universitat Ramon Llull, Facultat de Psicologia, Ciències de l’Educació i de l?Esport Blanquerna, Cister, 34, Barcelona, Spain; 4 Universidad Nacional de Educación a Distancia (UNED), Facultad de Psicología, Juan del Rosal, 10, Madrid, Spain; 5 Hospital de Mataró, Consorci Sanitari del Maresme, Carretera Cirera s/n, Mataró, Spain; 6 Capio Hospital General de Catalunya, Carrer Pedro i Pons, 1, Sant Cugat del Valles, Spain; 7 Parc de Salut Mar, CSMA Martí Julià, President Lluís Companys, 8, Santa Coloma, Spain; 8 CSM Nou Barris, Passeig Valldaura, 214 Baixos, Barcelona, Spain; 9 Hospital de la Santa Creu i Sant Pau, Sant Antoni Ma. Claret, 167, Barcelona, Spain; 10 Universidade do Minho, Escola de Psicologia, Campus de Gualtar, Braga, Portugal; 11 Department of Clinical Psychology and Psychotherapy, University of Bern, Gesellschaftsstrasse 49, Bern, Switzerland; 12 Department of Psychology, College Lane, University of Hertfordshire, Hatfield, Hertfordshire, UK

**Keywords:** Depression, Cognitive therapy, Implicative dilemmas, Cognitive conflicts, Personal constructs, Repertory grid, CORE-OM, Randomized clinical trial

## Abstract

**Background:**

Depression is one of the more severe and serious health problems because of its morbidity, disabling effects and for its societal and economic burden. Despite the variety of existing pharmacological and psychological treatments, most of the cases evolve with only partial remission, relapse and recurrence.

Cognitive models have contributed significantly to the understanding of unipolar depression and its psychological treatment. However, success is only partial and many authors affirm the need to improve those models and also the treatment programs derived from them. One of the issues that requires further elaboration is the difficulty these patients experience in responding to treatment and in maintaining therapeutic gains across time without relapse or recurrence. Our research group has been working on the notion of cognitive conflict viewed as personal dilemmas according to personal construct theory. We use a novel method for identifying those conflicts using the repertory grid technique (RGT). Preliminary results with depressive patients show that about 90% of them have one or more of those conflicts. This fact might explain the blockage and the difficult progress of these patients, especially the more severe and/or chronic. These results justify the need for specific interventions focused on the resolution of these internal conflicts. This study aims to empirically test the hypothesis that an intervention focused on the dilemma(s) specifically detected for each patient will enhance the efficacy of cognitive behavioral therapy (CBT) for depression.

**Design:**

A therapy manual for a dilemma-focused intervention will be tested using a randomized clinical trial by comparing the outcome of two treatment conditions: combined group CBT (eight, 2-hour weekly sessions) plus individual dilemma-focused therapy (eight, 1-hour weekly sessions) and CBT alone (eight, 2-hour group weekly sessions plus eight, 1-hour individual weekly sessions).

**Method:**

Participants are patients aged over 18 years meeting diagnostic criteria for major depressive disorder or dysthymic disorder, with a score of 19 or above on the Beck depression inventory, second edition (BDI-II) and presenting at least one cognitive conflict (implicative dilemma or dilemmatic construct) as assessed using the RGT. The BDI-II is the primary outcome measure, collected at baseline, at the end of therapy, and at 3- and 12-month follow-up; other secondary measures are also used.

**Discussion:**

We expect that adding a dilemma-focused intervention to CBT will increase the efficacy of one of the more prestigious therapies for depression, thus resulting in a significant contribution to the psychological treatment of depression.

**Trial registration:**

ISRCTN92443999; ClinicalTrials.gov Identifier: NCT01542957.

## Background

Depression is an often chronic and recurring health problem, with relapse rates ranging between 50 to 80% and approximately 120 million people currently affected worldwide. An increase in this figure is expected [1], and its lifetime prevalence can be up to 15% [2] - this constituting the leading cause of years lived with disability. In 2001, depression was the fourth main cause of morbidity and estimates indicate that, by 2020, it could rise to second place, after cardiovascular diseases [3,4]. Nowadays, depression is considered to present a predominantly chronic evolution with recurring episodes, and it attains such levels of severity that seriously compromises one’s quality of life. Furthermore, depression’s comorbidity with other mental health problems [5] and with other chronic diseases is very high, and worsens the chance of recovery [2], which further emphasizes the need to advance in the understanding and treatment of depression.

It is estimated that in Spain only 35.8% of patients diagnosed with major depression receive the minimum adequate treatment [6], but even amongst these, complete remission of symptoms is rare. In addition to drug treatments, a variety of existing psychological treatments have proved at least as effective for unipolar depression, without any single one of them standing out as more efficacious. However, cognitive behavioral therapy (CBT) has acquired more prestige and is backed by more research in efficacy [7,8], without this resulting in a fully satisfactory solution for the treatment of depression. Another issue raised by Haby and colleagues [9] is that the said efficacy cannot be asserted on the basis of existing research in languages other than English. Certainly, efficacy studies in Spanish are scarce and have very small samples.

Some of these problems in treating depression may be due to the fact that “that which we call ‘depression’ covers different problems and processes” [10]. Perhaps, despite the enormous amount of research and literature on the cognitive factors involved in depression [11,12], we need new conceptual advances ready to be tested in order to enrich cognitive models of depression. In particular, contributions that allow a better explanation of the difficulties (resistance, relapse, recurrence) encountered in the process of change are needed, difficulties which are also common in other mental disorders or health problems. Thus, these contributions might also be useful for the understanding and treatment of a variety of clinical conditions in which change is difficult to achieve.

Since its inception [13], cognitive therapy for depression was based on the identification of a systematic and persistent negativity in the cognitive processes of depressed patients. Thus, the identification of the negative thoughts which invade the patient’s consciousness in an automatic fashion is central to this therapeutic approach. Dysfunctional schemas, attribution bias, rumination and perfectionism are also part of the cognitive model for depression, amongst other factors [14]; however, as Beck himself recently pointed out [15,16], the formulated model is incomplete and needs to be supplemented.

One aspect which has not been studied, and which could contribute to explaining the difficulties of these patients achieving change, concerns the cognitive processes related to identity conflicts. Our research group has been working for years with a methodology capable of assessing these conflicts and performing empirical studies to evaluate its clinical importance.

### The notion of cognitive conflict

Starting with psychoanalysis, a variety of theories have suggested the existence of internal conflicts which drive people to grueling internal struggles blocking their development, and creating suffering and symptoms. Heider and Festinger’s now classic formulations stand out, which postulated a motivational tendency to resolve these conflicts, and how, when this is not attained, internal conflicts generate a state of psychological tension. Piaget himself [17] proposed the term “cognitive conflict”, which in a developmental context serves the function of reorganizing the intellectual processes of children. Unfortunately, and despite the enormous relevance of this notion, it has received little attention in recent decades, largely due to the lack of methods providing operational and measurable definitions.

Personal construct theory (PCT) provides an ideal conceptual and methodological framework for the study of cognitive conflicts. Far from being anchored in the rich initial formulations of its creator [18], PCT has undergone considerable development (see, for example, Fransella’s extensive compilation [19] or Walker and Winter’s review [20]). PCT emerges from a proactive view of human beings as active agents (as later did Bandura [21]) who regulate their motivational and emotional processes as well as their actions, based on the congruence or discrepancy between their construction of “self” and “ideal self” (as suggested also by other authors [22-25]. Thus, it is understandable for the person to encounter dilemmas or conflicts when having to reconcile themselves with their personal values in a decision-making process (although the person might not be consciously aware of those conflicts).

Rowe [26] describes the case of a chronically depressed patient who faced the choice between staying depressed (linked in her system to being “humane”), or improving her mood but becoming a “destructive” or “unpleasant” person (according to her own view). In PCT, the notion of implicative dilemma emerged to designate those conflicts in which the desired change (for example, stop being depressed) implies, in the context of the network of constructs forming a personal meaning system, undesired changes in one’s vision of oneself (for example, becoming “unpleasant”). When these conflicts occur in the construct system of an individual, his or her capacity for change becomes blocked, which would be relevant to explain both the onset of clinical symptoms as well as the resistance to treatment. Although these cognitive conflicts can coexist with interpersonal conflicts and with secondary gains as postulated by other theoretical approaches, they are independent from these and represent an innovative contribution of PCT.

The repertory grid technique (RGT) [27,28] stems from PCT as an instrument that allows the self-concept and its cognitive structure to be assessed. The main advantage of this technique is that it is based on the evaluation made by the person themselves and others through their own constructs or mental contents, including what they consider their “ideal self” (see Method section for more details). It is therefore a subjective assessment technique [29], with high levels of utilization (see [30]), which is ideal to capture conflicts in personal meaning systems. The RGT has already been applied in the study of unipolar depression (for example, [31-33]; see Winter [34] for a review).

**Figure 1:**
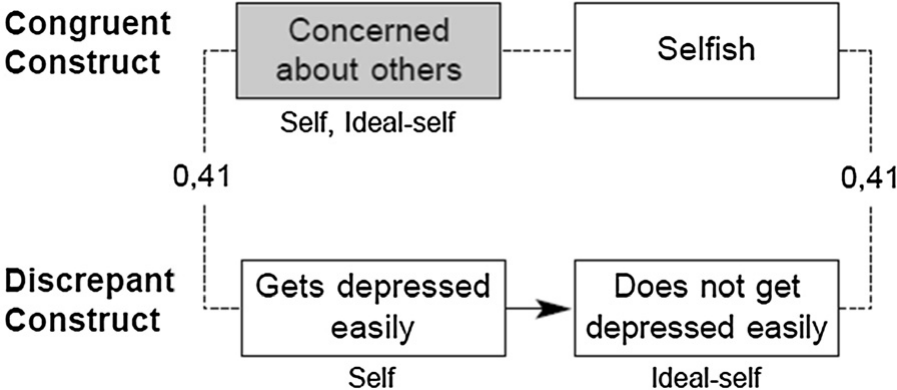
**Implicative dilemma found in a grid of a patient with depressive symptoms.** From [36]. Reproduced with permission.

One of our group’s contributions is the development of a means to evaluate conflicts in relationships between constructs, operationalizing the notion of implicative dilemma (ID) through the RGT [35,36]. In the first step of our procedure, discrepant constructs are identified when scores between the “self” and “ideal self” differ. In the second, congruent constructs (in which there is similarity between “self” and “ideal self”) are also identified. Next, correlations between both groups of constructs are analyzed in pairs, and implicative dilemmas are detected in those cases where the desirable pole of the discrepant construct (for example, “does not get depressed easily”) correlates with the undesired pole of the congruent construct (for example, “selfish”). Figure 1 offers an example of this ID. In the construct system of the person depicted in the example, overcoming depression in a consistent way would also imply becoming selfish. Also, to make sure they are really “concerned about others”, they must remain depressed. So the person is immersed in a dilemma because of the implication that the discrepant construct has for the congruent construct (and *vice versa*).

Cognitive conflicts might be reflected in the RGT also by the use of dilemmatic constructs, those in which the “ideal self” is rated at midpoint. This might indicate that both poles of the construct designate undesirable characteristics for the self, the person thus trying to regulate their position in the construct by avoiding the extremes.

### Cognitive conflicts, depression and psychotherapy

Preliminary studies with this definition of cognitive conflicts understood as dilemmas have shown their clinical interest. Feixas and collagues [37] found that just over half of a group of patients who consulted for psychotherapy (n = 284) presented with IDs, while only a third of the control group did (n = 322). This statistically significant difference was not found with two other procedures used to identify conflicts in the RGT. In addition, participants with IDs had a higher level of symptom severity. Feixas and colleagues [38], in a pilot study, found a higher proportion (59.60%) of depressed patients with at least one ID (versus 40.40% of the control group). Also in this study, symptom levels were associated with the number of these dilemmas. In a presentation of preliminary results of an ongoing project with 81 depressive patients and improved reliability of the RGT application, Feixas and colleagues [39] found that although cognitive conflicts (including both IDs and dilemmatic constructs) were not specific to depression, they occurred in about 9 out of every 10 patients.

Regarding the effects of psychotherapy on IDs, Feixas and colleagues [40] followed a group of 87 patients and found that those who presented these conflicts to begin with tended to resolve them by the end of psychotherapy (although psychotherapy had a variety of orientations and did not focus on dilemma resolution). Moreover, in cases where this did not happen, there was less symptomatic improvement. This leads us to formulate the desirability of addressing these conflicts directly in psychotherapy as a way of enhancing its efficacy. To this end, our group has developed a manual of dilemma-focused therapy (DFT) published in English [41], Spanish [42] and Portuguese [43]. However, experience and data accumulated so far do not lead us to conceive DFT as enough for a comprehensive treatment for depression. The presence of dilemmas is not postulated as a causative agent or as the only maintenance factor of depression (or of any other disorder), but rather as a cognitive structure that may hinder the process of change. For this reason, we do not conceive treatment of depression as using exclusively DFT, but rather propose DFT as an additional component to a broader treatment as a way to enhance efficacy.

### Justification and main hypothesis

The notion of mental or intrapsychic conflict has been discussed for over a century by multiple theoretical approaches, but it has not been defined in measurable operational terms. The importance of said conflicts with regard to depression has not been described either. Based on PCT, we have developed a procedure to identify IDs, a type of cognitive conflict, using RECORD 5.0 ([44]; the English version GRIDCOR 4.0 is also available [27]), an application software for the analysis of RGT data. Preliminary results [39] indicate that about 90% of these patients have at least one of the cognitive conflicts (dilemmas) as formulated by our team based on their repertory grids. These results justify the development of a manualized intervention, DFT, to resolve these conflicts [41-43].

The present study aims to adapt DFT to a specific individual intervention for unipolar depression combined with CBT in a group format, the effectiveness of the latter having been already demonstrated [45]. In order to assess the efficacy of this combined package, it will be compared to patients receiving CBT both in group and individual format. If this new form of combined therapy (group CBT plus individual DFT) exceeds the efficacy of the condition using only CBT (group plus individual), the project will have provided a potential improvement which, if applied, would produce significant benefits to depressive patients and, consequently, for society in general. The reason for selecting CBT as the comparison group is the well-established level of efficacy of this therapy for depression, both in individual and group formats [46]. Thus, this trial intends to test the hypothesis that the design of an intervention module focused on the dilemmas identified in each patient can contribute to improving the efficacy of cognitive therapy for depression.

### Specific objectives

• To study whether or not a combined therapy (group CBT plus individual DFT) is more efficacious than CBT applied in both group and individual formats.

• To check whether the degree of resolution of cognitive conflicts (dilemmas) is greater in the treatment condition specifically centered on dilemmas in comparison to the well-established CBT control treatment.

• To identify the pretreatment variables that predict the outcome of the two treatment conditions studied, in particular diagnosis (major depressive disorder (MDD), dysthymic disorder (DD)), chronicity, severity and cognitive indicators derived from the RGT.

### Pilot studies

Prior to the final version of this research protocol, two brief non-randomized studies were conducted consecutively to explore the best combination of group CBT and individual therapies (CBT and DFT) in terms of clinical applicability. Each study consisted of a small cohort of patients receiving a particular combination of these therapy formats. In the first study, after initial assessment (T1) two therapists conducted 10 group CBT sessions. Then, a new assessment (T2) took place before the next phase of individual therapy (10 sessions of CBT, or DFT for those patients with cognitive conflicts) carried out by different therapists. In the post-therapy assessment (T3) clients were interviewed in depth about the usefulness and convenience of the format of therapy received. They considered it helpful but mostly found the intermediate assessment in between group and individual phases to be an interruption of the rhythm of treatment. In discussing the experience with therapists and supervisors it was also estimated that both phases could be shortened to eight sessions without major loss. A second study was then conducted with another small cohort receiving a format of eight group CBT sessions plus eight individual (CBT or DFT) sessions without the intermediate assessment. In this case, patients suggested that the continuity with the therapist from group to individual sessions would be more convenient. These pilot studies were useful not only for the better adjustment of the design with respect to the combination of the group and individual therapy formats, but also for the adaptation of the therapy manuals to be used in the main study.

## Method

### Design

**Figure 2:**
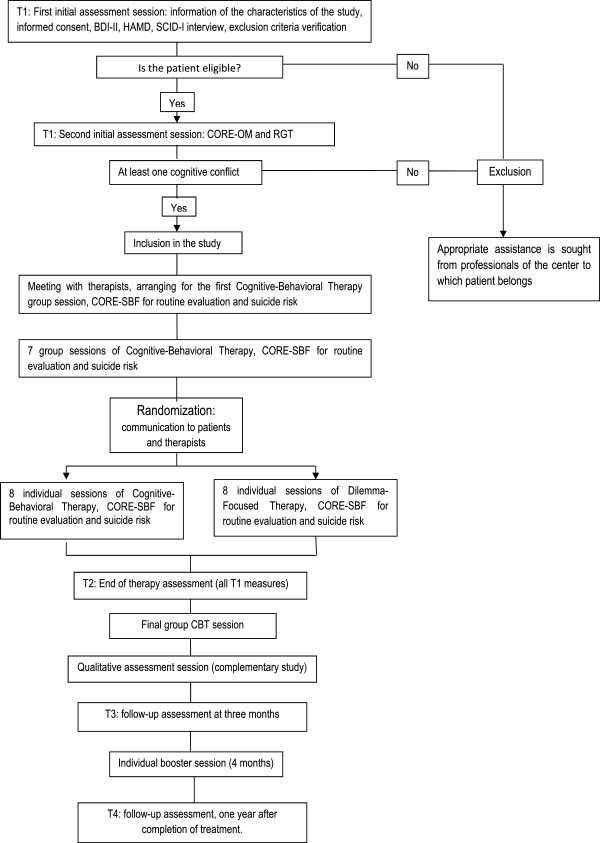
**Clinical Study Design.** BDI-II, Beck depression inventory, second edition; CBT, cognitive-behavioral therapy; CORE-OM, clinical outcomes in routine evaluation-outcome measure; HAMD, Hamilton rating scale for depression; RGT, repertory grid technique; SCID-I, structured clinical interview for DSM-IV axis I disorders; CORE-SFB, short form B of the clinical outcomes in routine evaluation-outcome measure.

This study follows a controlled trial methodology to evaluate the efficacy of treatments. Because DFT is not conceived as a whole treatment for depression, but rather as an ingredient which can be added to other therapies, the study is designed to test the efficacy of a combined package which includes DFT as a substantial component. As a comparison group we use CBT for depression, a treatment package with a well-established effectiveness both in individual and in group formats. Since DFT so far can only be applied in the individual format (because it focuses on the dilemmas identified specifically for each patient), group CBT is included as a shared part of both treatment conditions. This treatment modality has already been proven effective (to levels comparable to individual CBT format) and also more efficient in terms of treatment costs. In summary, all patients receive group CBT, and added to this condition is a component of individual therapy which, depending on randomization, can be CBT or DFT. For each treatment condition a therapy manual has been adapted for the specific format of the study. The design of the clinical trial is depicted in Figure 2.

### Setting

The study will be conducted at various mental health centers in the Barcelona area: *CSM Nou Barris*, *Associació Catalana de Teràpies Cognitives* (ACTC) and others belonging to *Hospital de Mataró*, *Parc de Salut Mar* (Santa Coloma), and to *Fundació Sant Pere Claver*. Furthermore, patients will be recruited from primary care centers and treated either in those centers or at ACTC.

### Participants

Since this is the first controlled study of DFT, no previous data are available on which to base our estimations. However, according to other studies comparing CBT with a variety of other therapies (see the meta-analysis by Gloaguen and colleagues [47]), we estimated medium between-group effects. Thus, to determine the sample size we calculated that, with a statistical power of 0.80, considering a dropout rate of 20%, an α significance level of 0.05 (two-sided) and an effect size of 0.30, we require a total of 112 patients. In terms of our primary outcome measure (Beck depression inventory, second edition (BDI-II), see below) and based on Spanish normative data, calculation yielded a 3.3-point difference between groups.

#### Inclusion criteria

All participants will meet diagnostic criteria for MDD or DD according to the *Diagnostic and Statistical Manual of Mental Disorders* (4th edition, text revision) (DSM-IV-TR) [48] and a score above 19 on the BDI-II questionnaire. Once the RGT is administered only those patients who have at least one cognitive conflict are included in the treatment phase of the study, which based on previous data [39] should be 90% of the cases. The age range is between 18 and 70 years.

#### Exclusion criteria

Patients presenting bipolar disorders, psychotic symptoms, substance abuse, organic brain dysfunction, acute suicidal ideation or mental retardation will be excluded from the study. Those who are receiving psychological treatment will not be accepted either, unless it is suspended at the time of inclusion in the study itself, in agreement with the patient and the therapist who is applying it. The presence of other comorbid conditions (anxiety, eating or personality disorders, etc.) and consumption of psychotropic drugs will not be cause for exclusion but they will be assessed and recorded for statistical control. Finally, those who do not have enough level of competence to communicate in Spanish or Catalan, or patients with substantial visual, hearing and cognitive deficits cannot be included.

### Instruments

– Structured clinical interview for DSM-IV axis I disorders (SCID-I) [49] for the diagnosis of mental disorders and collection of socio-demographic data, psychotropic drug consumption, and treatments received.

– BDI-II [50,51], a 21-item self-report instrument to assess the existence and severity of symptoms of depression. It has good psychometric properties and acceptability. This is the main outcome measure.

– Clinical outcomes in routine evaluation-outcome measure (CORE-OM) [52], a 34-item self-report questionnaire for the assessment of subjective well-being, symptoms or problems, life functioning and risk. It has good psychometric properties and it has been adapted into Spanish for free distribution recently [53]. The short form B of the clinical outcomes in routine evaluation-outcome measure (SFB) of 18 items will be used for session-to-session monitoring of the therapy process.

– Hamilton rating scale for depression (HAMD) [54] is the most widely used outcome measure in clinical trials for depression. It is a clinician-rated 17-items instrument with good psychometric properties.

– RGT [27] for assessing the presence, number and severity of cognitive conflicts. It is a semi-structured interview oriented at the elicitation of personal constructs and their assessment as they are applied to a set of significant others (for a more detailed description of the format used in this study see [36]). Other repertory grid measures assessing the construction of self and cognitive structure will be examined in another study.

### Treatments

#### Group cognitive behavioral therapy

A comprehensive therapy manual [Bados and García-Grau, *Terapia cognitivo-conductual de la depresión: Manual de tratamiento en grupo.* Universitat de Barcelona. Unpublished manuscript] has been created for this study based in Beck’s cognitive therapy manual [13] and two more recent publications [55,56]. The manual has been adapted to eight group sessions of approximately 120 minutes in length. This treatment will be applied to all patients in the study, led by two therapists, both licensed psychologists with sufficient experience and training to carry it out. The groups will be formed with 6 to 8 patients, and thus it is estimated that between 14 and 18 therapy groups will be conducted. Furthermore, once the seventh session is finished, patients will receive (following randomized allocation) one of the two types of individual psychotherapy compared in this study (CBT or DFT) by the same group therapists (one in each condition). Once this phase of individual sessions is done, another group CBT session lasting 180 minutes (with a break) will be scheduled. This final session, focused on relapse prevention, is thought to also provide a sense of continuity for all the therapy process and reunion of all patients and therapists involved.

#### Individual cognitive behavioral therapy

Patients assigned to this condition will receive CBT following a complementary manual [Bados and García-Grau, *Terapia cognitivo-conductual de la depresión: manual de tratamiento individual.* Universitat de Barcelona. Unpublished manuscript] based on the same sources as above. The manual has been adapted to eight sessions of approximately 60 minutes and to the fact that patients have received group CBT previously. The therapist will also have conducted that group.

#### Individual dilemma focused therapy

Patients assigned to this condition will receive DFT following the manual designed specifically for this project [Feixas and Compañ, *A dilemma-focused intervention for unipolar depression: individual treatment manual.* Universitat de Barcelona. Unpublished manuscript], based on Feixas and Saúl [41] and Senra and colleagues [42]. Since this type of intervention has never been tested before, the process for creating this manual followed the indications of Carroll and Nuro [57] for the development of therapy manuals. The therapy will consist of eight sessions of approximately 60 minutes.

In DFT manuals (and in very general terms), the first session comprises welcoming and establishing rapport, analysis of demand, therapy goals definition, and identification of main dilemmas. Subsequently, feedback of the results of the evaluation is provided and the presenting problem(s) or symptom(s) is reframed in terms of the dilemma(s). Next, work on the dilemma(s) is conducted over several sessions, and the therapeutic process is completed with the revision of the work done on dilemma(s) and future prospects. During all phases, techniques originating from personal construct therapy are used (for example, see [58]). As in the CBT condition, the therapist will have previously conducted group therapy with these patients.

### Procedure

This study was approved by the Committee for Ethics in Research of the University of Barcelona under the number (IRB00003099) and also by the ethical committees of the other centers involved in the study. It is registered at ClinicalTrials.gov under number NCT01542957 and also at Current Controlled Trials (ISRCTN92443999).

Professionals of collaborating centers will be asked to inform their patients with major depression or dysthymia of the existence of this psychological treatment study and enable us to contact those who accept the opportunity to participate. For each center, an information sheet for potential participant patients and another for professionals with information on how to refer patients for the study were created. Finally, to reach a wider population range, a web page (http://www.ub.edu/terdep) and a flyer have been produced for the study. After referral by telephone or email, patients will be contacted to arrange for the selection and assessment process.

#### Initial assessment

The initial assessment (T1) will be carried out in three phases. Firstly, the nature of the study is explained and, if the patient agrees to participate and signs the informed consent, the BDI-II is applied to verify the clinically significant presence of depressive symptoms (a score above 19). Secondly, patients meeting this criterion will be evaluated using the SCID-I interview to confirm the diagnosis of MDD or DD (if this is not the case, they will be excluded) and explore for other comorbid disorders. The section “Overview” included in this interview will also be used to collect socio-demographic data and information relative to previous treatments, to clarify that they are not receiving and will not receive any other concurrent psychological treatment, and to record psychotropic drug consumption. A few other instruments will also be used in this and further assessment sessions as part of another study. With patients selected in this phase, a second assessment session will be scheduled in the following weeks. In this third phase, the CORE-OM and the RGT will be administered. The resulting data will be analyzed with the RECORD 5.0 software [27,44]. On the following day, they will be contacted to confirm their inclusion in the study (if they have at least one cognitive conflict) or exclusion (in which case a way of providing the appropriate assistance to them will be sought together with the professionals from the center where they came from). The evaluators will be psychology graduate students (or advanced undergraduates) who will have been specially trained to perform the entire assessment process, as in particular the SCID-I and the RGT interviews require specific training, and they will be, at this point, blind in regard to the treatment modality which will be assigned to the patients. Two evaluators will conduct the assessment process for each potential participant.

#### Beginning of the treatment and randomization

Once assessed and accepted for the study, patients will be summoned individually to a meeting with their therapists to explain the psychological treatment combining group and individual therapy, and then convened for the first CBT group session. In this meeting and at the beginning of the consecutive 16 therapy sessions, the CORE-SBF will be administered for routine evaluation of the therapy process and to monitor suicide risk. Whenever patients rate 3 or 4 on item 5 (suicide plans) they will be given special attention after the group session for a more careful assessment of suicide risk as specified in the therapy manual.

Once the first phase of seven group sessions is finished, they will be randomly assigned to one of the two individual treatment conditions (CBT or DFT) which will begin following the group therapy. Randomization will be done using permuted blocks with a random assignment online application [59] by a staff member of the Department of Personality, Assessment and Treatment of the University of Barcelona, completely oblivious to the study and blind to treatment conditions. Therefore, group CBT therapists can only know of patient allocation after the first phase of group treatment is completed, right at the moment they have to start individual therapy. In any case, therapists will not have participated in the initial evaluation of those patients nor will they in any subsequent assessments.

Treatments will be carried out mostly in the health centers corresponding to the patients according to the Catalan Public Health System, both in the common group format and in the two individual treatment conditions. If, eventually, the treatment cannot be carried on in these centers, patients will be invited to participate in the groups of ACTC. Groups will be formed in the various centers as soon as 8 patients have been admitted to the study and agree to initiate the proposed treatment.

Therapists will be graduate students enrolled in the master in Cognitive Social Therapy at the University of Barcelona. This is not a research-oriented course but rather a 3-year program designed to provide therapy training. Both group and individual sessions will be recorded on audio (which is also reflected in the informed consent). This will allow supervision of each session from members of the team with more clinical experience who will evaluate the performance of the therapists in order to maximize their adherence to the relevant manual and ensure that therapy is beneficial for its recipients. Before the start of the treatment, differentiated training workshops will be developed for each type of treatment to provide therapists with specific training in the protocols for individual and group therapy used in the study.

#### Consecutive assessments, termination and follow-up

Once the individual therapy phase is terminated, a second assessment (T2) of all initial measures will be carried out by the same pair of evaluators who conducted the initial assessment (whenever possible), blind to the therapeutic condition of each patient. In the following week the final group session will take place and a more qualitative assessment session (as part of a complementary study) will take place in another week. There will also be follow-up assessments at 3 months (T3) and 1 year (T4) after completion of treatment to check the degree of maintenance of the accomplished changes. Finally, one (or occasionally two) booster sessions will be offered to all patients during the fourth month after treatment termination.

#### Treatment integrity

For group CBT and the individual CBT arm of the study, therapist adherence will be assessed using the revised cognitive therapy scale (CTS-R) [60]. Because there are no pre-existing adherence/fidelity measures for DFT, a scale will be created to measure therapist adherence to this individual therapy arm.

Two trained clinical graduate students will independently rate a random selection of sessions using the CTS-R for the group sessions and both scales for the individual ones. Inter-rater reliability and success at discriminating between the content of the CBT and DFT will be computed. Patient’s treatment compliance will be monitored by attendance and homework completion.

### Statistical analyses

All data derived from the measurements and the clinical data (including use of psychotropic drugs) and socio-demographic information will be entered into Statistical Package for Social Sciences version 20.0 or later (IBM Corporation, Armonk, New York, USA). The main statistical calculations will be performed on an intention-to-treat basis (patients will be analyzed as initially randomized). Categorical data will be summarized as frequencies and percentages. Means and standard deviations will be used for continuous data for which normality tests will also be performed.

Analyses will include elementary head-to-head comparisons of the intervention groups as well as mixed models of repeated measures analysis of variance in which initial measures (baseline) of the dependent variables will be used as covariates. Sociodemographic and clinical differences between patients in response to treatment (such as diagnosis of MDD or DD, and chronicity) will be explored, as well as the potential mediator or moderator role of the cognitive measures obtained with the RGT, especially in regard to the number and intensity of cognitive conflicts. In fact, additional analysis will take these conflicts as a dependent variable to observe how they have varied with the different treatments. Also as secondary analyses, the inclusion of the CORE-OM/SFB in the study will allow the examination of the degree of therapeutic change in other aspects, such as the patients’ psychosocial functioning and subjective well-being. Moreover, it will also allow assessment of the change experienced in relation to the number of sessions and the percentage of change per session. In all the analyses which permit it, and depending on the variations found between the groups, patient drug consumption will be statistically controlled. In all statistical contrasts, derived confidence intervals will be taken into account as well as their effect sizes.

Finally, we will calculate the proportion of patients with a reliable and clinically significant improvement on the BDI-II, HAMD-17 and CORE-OM outcome measures. We will follow Jacobson and Truax [61] who established that clinical improvement is based on both reliable change (the extent to which pre-to-post-difference scores are reliable) and on clinical significant change (the extent to which post-treatment scores are clinically meaningful). Chi-square tests will be used to test the frequency differences in these categories between the two intervention groups.

## Discussion

Despite advances in its prevention and treatment, depression is nowadays one of the most serious health problems we encounter, both for its morbidity as well as its disabling effects. This is a problem with major implications on the lives of those affected and their families, as well as a major social and health burden. Most cases do not fully remit and, for those who do, recurrence is very common. This persistence (sometimes intermittent) of the depressive discomfort is one of the most difficult issues to resolve in the field.

Cognitive models have made a significant advance in the understanding of depression and have come a long way since initial formulations. Thus, therapeutic targets (for example, activity scheduling, identifying automatic thoughts, etc.) emerged and were identified to guide CBT. However, only partial progress has been accomplished so far in the treatment of depression. The existence of conflicts or dilemmas in the cognitive structure is more prevalent in depression, and could explain the blockage and relapses that characterize these patients, especially the more severe and/or chronic. Our hypothesis is not that these cognitive conflicts are the cause or the main factor maintaining the depression, but rather we believe the dilemmas commonly found in depressive patients [38,39] might reveal that their discomfort is congruent with a part of their cognitive system (while for another part the change is desirable). If these dilemmas are identified they should become a focus of the psychological intervention so as to not hinder the process of change. In cognitive-behavioral treatment of depression, cognitive conflicts may go unnoticed in many cases, or may not receive sufficient attention. Therefore, this study aims to test whether the addition of a specific module focused on working with dilemmas can result in a significant increase of the efficacy of cognitive therapy for depression. Presumably this combined intervention will benefit to a greater extent those patients who are more severe or with a higher chronicity, with the personal, social and economic consequences that this condition entails.

This particular design, however, does have some limitations. Even if results show the superiority of the DFT arm, their success cannot be attributed only to DFT but to its combination with group CBT. Conversely, if the arm including DFT does not show better outcome results than the comparison CBT package, this would not rule out the possibility that individual DFT could be effective. In fact, it could be effective in combination with other treatments. Still the design of the study can be informative as a first step in testing the relative efficacy of DFT in terms of pursuing further investigation with this treatment modality. Also, the fact that treatments are applied by novice therapists might lessen the potential effects of the treatments being tested. For that purpose, as said, clinical supervision is carefully provided in the study but that might not be enough for the treatments to be applied with a high level of competence. An additional limitation of the study is that, although we plan to collect up to 1-year follow-up data, a longer period might be more convenient for properly considering relapse and recurrence as outcome variables.

In addition to the main goal of this study, other additional benefits might also be attained with the implementation of this study. First, the development and adaptation of therapy manuals eases the dissemination of these psychological treatments amongst professionals working in Spanish and their further application in clinical practice. Moreover, they provide a useful tool for training novice therapists, improving the competence of more experienced therapists and facilitating the development of new empirical studies. Since DFT will have been applied by relatively novice therapists (graduate students in training), it is expected that the adoption of this treatment by more experienced professionals will not be difficult. However, further investigation should be made to study whether more seasoned therapists who had been working in clinical practice for years find the DFT manual feasible to learn and to apply it with their patients. Second, it will contribute to provide further evidence in Spain of CBT for depression in general, regardless of whether intervention with dilemmas adds value to them or not. Indeed, there are few controlled studies with sufficiently large sample sizes in our country or in languages other than English [9], and thus the effectiveness of these therapies needs to be confirmed. Finally, this will be the first randomized clinical trial using CORE-OM in Spanish, which also contributes in this regard. As an instrument specializing in measuring therapeutic change used in many public and private health centers in the UK, its use in this study will confirm its usefulness also in Spanish.

Two of the authors of this study are recognized CBT experts and trainers while a few others are well known in the constructivist therapies. The inclusion in the team of researchers with different theoretical orientations and closely linked to the two types of intervention modalities that are subjected to empirical testing aims to control for an allegiance effect of the researcher on the study results [62]. Another advantage of the present team composition is the inclusion of international experts on psychotherapy research and on DFT/constructivist therapy who act as consultants for the entire research process.

In case of favorable outcome it would be convenient for other independent research groups to replicate this study so that the combination of group CBT and individual DFT may be considered an efficacious and specific treatment. Moreover, further studies should address the limitations of controlled studies, especially in regard to its generalization and dissemination among clinicians. However, this study will be implemented in a way that will secure high levels of generalizability. On the one side, most patients will be recruited and treated in their primary care or mental health centers. On the other, therapists’ level of experience will be relatively low so the impact of the therapy manuals might be more substantial. To potentiate this factor, supervision and careful analysis of therapists’ adherence to the manual will be performed by experienced therapists. Therefore, results of the study should be generalizable to novice therapists working in naturalistic settings.

Future studies might also explore whether this DFT module increases the efficacy of not only CBT but also of other treatments for depression such as interpersonal therapy or emotion-focused therapy. In all these possible studies, cost-benefit analysis would be informative as well.

## Trial status

Recruitment of participants is ongoing; it began in November 2011 and is expected to end in June 2014.

## Abbreviations

ACTC: *Associació Catalana de Teràpies Cognitives*; BDI-II: Beck depression inventory, second edition; CBT: cognitive-behavioral therapy; CORE-OM: clinical outcomes in routine evaluation-outcome measure; CTS-R: revised cognitive therapy scale; DD: dysthymic disorder; DFT: dilemma-focused therapy; DSM-IV-TR: Diagnostic and Statistical Manual of Mental Disorders (4th edition, text revision); HAMD: Hamilton rating scale for depression; ID: implicative dilemma; MDD: major depressive disorder; PCT: personal construct theory; RGT: repertory grid technique; SCID-I: structured clinical interview for DSM-IV axis I disorders; SFB: short form B of the clinical outcomes in routine evaluation-outcome measure

## Competing interests

The authors declare that they have no competing interests.

## Authors’ contributions

AB, EGG and GF participated in the design of the study and coordination of the entire project. AM, GD, LB, VC, JMS, LASG, MA and SC participated in the design of the study. GF, AB, EGG, MS and VC participated in the pilot studies. CP, MS and AT helped to draft this manuscript. JMS and AB participated in planning the statistical analysis. JC, MG, MI, LMF and JS made substantial arrangements for the recruitment of participants and data collection. ER, FC and DW critically reviewed the whole project. GF is the principal investigator, conceived of the study, participated in its design and coordination with participating centers and drafted the manuscript. GF and AB took responsibility for incorporating suggestions in the final version. All authors read, made suggestions and approved the final manuscript.
